# Sleep fragmentation frequency is associated with distinct immune, microbial, and barrier-related profiles in DSS-induced colitis in mice

**DOI:** 10.3389/fimmu.2026.1885856

**Published:** 2026-09-15

**Authors:** Ming-Shih Tu, Ting-An Fan, Yueh-Hsiang Huang, Ren-Chin Wu, Chia-Jung Kuo, Ning-Hung Chen, Li-Pang Chuang, Shih-Wei Lin, Yen-Lung Chen, Huang-Yu Yang, Geng-Hao Liu

**Affiliations:** 1 Graduate Institute of Traditional Chinese Medicine, School of Traditional Chinese Medicine, College of Medicine, Chang Gung University, Taoyuan, Taiwan; 2 Center for Traditional Chinese Medicine, Chang Gung Memorial Hospital, Taoyuan, Taiwan; 3 School of Traditional Chinese Medicine, College of Medicine, Chang Gung University, Taoyuan, Taiwan; 4 Division of Chinese Internal Medicine, Center for Traditional Chinese Medicine, Chang Gung Memorial Hospital, Taipei, Taiwan; 5 Department of Anatomic Pathology, Linkou Chang Gung Memorial Hospital, Taoyuan, Taiwan; 6 Department of Gastroenterology and Hepatology, Linkou Chang Gung Memorial Hospital, Taoyuan, Taiwan; 7 Sleep Center, Chang Gung Memorial Hospital, Taoyuan, Taiwan; 8 Department of Pulmonary and Critical Care Medicine, Linkou Chang Gung Memorial Hospital, Taoyuan, Taiwan; 9 Division of Acupuncture and Moxibustion, Center for Traditional Chinese Medicine, Chang Gung Memorial Hospital, Taoyuan, Taiwan; 10 Department of Nephrology, Linkou Chang Gung Memorial Hospital, Taoyuan, Taiwan; 11 School of Medicine, College of Medicine, Chang Gung University, Taoyuan, Taiwan; 12 Johns Hopkins Bloomberg School of Public Health, Baltimore, MD, United States

**Keywords:** epithelial barrier, gut microbiota, microbiota-derived metabolites, short-chain fatty acids, sleep fragmentation, ulcerative colitis

## Abstract

**Background:**

Sleep fragmentation is common in inflammatory bowel disease, but whether different fragmentation frequencies are associated with distinct biological responses during colitis remains unclear. This study examined endpoint-specific responses to low- and high-frequency sleep fragmentation in DSS-induced colitis in mice.

**Methods:**

Male C57BL/6 mice were assigned to Control, DSS, DSS with low-frequency sleep fragmentation (DSS+LSF), DSS with high-frequency sleep fragmentation (DSS+HSF), or high-frequency sleep fragmentation alone (HSF). Colitis was induced with 2% DSS for 7 days. Sleep fragmentation was applied for 12 h/day during the light phase at 60 sweeps/h for LSF or 240 sweeps/h for HSF. Clinical indices, histology, epithelial barrier-associated proteins, inflammatory and stress-related markers, colonic CD4^+^ T-cell subsets, fecal microbiota composition, and fecal SCFAs were assessed.

**Results:**

Clinical and macroscopic indices varied across experimental groups, but corrected comparisons among DSS-treated groups were not significant. Plasma corticosterone was increased in both DSS+LSF and DSS+HSF, whereas MPO was higher in DSS+LSF than in DSS+HSF. Colonic Th2 proportions were higher in DSS+HSF than in DSS and DSS+LSF. Other histological, barrier-associated, inflammatory, and metabolic findings were primarily descriptive.

**Conclusions:**

Low- and high-frequency sleep fragmentation were associated with distinct, endpoint-specific response profiles during DSS-induced colitis rather than a simple linear increase in disease severity. These findings support frequency-associated, domain-specific responses and warrant further longitudinal and mechanistic investigation.

## 1 Introduction

Inflammatory bowel disease (IBD), encompassing ulcerative colitis (UC) and Crohn’s disease (CD), is a chronic relapsing inflammatory disorder thought to arise from complex interactions among genetic susceptibility, environmental triggers, gut microbiota, epithelial barrier dysfunction, and dysregulated mucosal immune responses (1). Despite advances in biologic and small-molecule therapies, symptom burden and recurrent flares remain common, highlighting the need to identify modifiable factors that shape intestinal inflammation and host resilience.

Sleep disturbance is increasingly recognized as a prevalent and clinically meaningful comorbidity in IBD. A recent systematic review and meta-analysis reported that poor sleep is present in more than half of patients with IBD, and its prevalence is higher with objective markers of disease activity (2). Importantly, sleep impairment in IBD is not solely attributable to nocturnal gastrointestinal symptoms; elevated systemic inflammation (e.g., high C-reactive protein) has been associated with poor sleep quality even after accounting for nighttime disruption (3). Objective measurements further suggest that sleep fragmentation—recurrent brief arousals that disrupt sleep continuity—can be observed even in patients with clinically inactive IBD (4). Together, these findings support a model in which inflammation and disrupted sleep may reinforce each other, with sleep quality representing a potentially modifiable factor in IBD management.

Epidemiologic and prospective cohort evidence also supports a bidirectional relationship between sleep and IBD. In a large longitudinal cohort of patients with IBD in remission, baseline sleep disturbance predicted a higher risk of subsequent active disease in CD (5). In addition, population-based prospective data indicate that both short and long sleep duration are associated with an increased risk of incident UC, suggesting that abnormal sleep may influence disease development as well as progression (6). These clinical observations motivate experimental studies to clarify how specific sleep phenotypes, particularly sleep fragmentation, might modulate intestinal inflammation.

A putative biological framework in IBD involves the interplay among gut microbiota dysbiosis, microbial metabolite alterations, epithelial barrier dysfunction, and mucosal immune activation. Longitudinal multi-omics profiling has demonstrated characteristic functional dysbiosis during IBD activity, including shifts toward facultative anaerobes and disruptions in microbial metabolite pools that include short-chain fatty acids (SCFAs) (7). In UC, reduced abundance of key butyrate-producing bacteria such as Roseburia hominis and Faecalibacterium prausnitzii has been reported, consistent with impaired SCFA-associated ecological functions (8). SCFAs (acetate, propionate, and butyrate) are increasingly recognized as important mediators linking diet–microbiota interactions to epithelial and immune homeostasis: they serve as energy substrates for colonocytes, support barrier integrity, and regulate innate and adaptive immune responses (9–11). Barrier impairment itself is not merely a consequence of inflammation; tight junction disruption can amplify mucosal immune activation and facilitate sustained inflammatory cascades in IBD (12). Consistent with this framework, sleep and circadian disruption have been reported to influence intestinal barrier function, microbial metabolite profiles, and experimental colitis severity, suggesting a plausible interface between sleep-related physiology and gut barrier–immune regulation (13, 14).

Sleep fragmentation is a distinct and clinically relevant sleep-disruption phenotype, commonly occurring in conditions such as obstructive sleep apnea, where recurrent arousals fragment sleep architecture (15). Experimental models indicate that fragmented sleep can alter the gut microbiome and fecal metabolome, including changes in putative SCFA-producing taxa (16, 17). Separately, animal studies have shown that disrupted sleep can worsen experimental colitis and delay recovery, underscoring the biological plausibility that sleep disruption can modulate intestinal inflammation (18). However, most preclinical work has compared a single sleep-disruption protocol against controls, leaving a critical gap in understanding whether different frequencies of sleep fragmentation are associated with distinct clinical, inflammatory, microbial, metabolic, and barrier-associated endpoint profiles during colitis.

To address this gap, we used a DSS-induced colitis model, a widely used model of UC-like epithelial injury and intestinal inflammation (19), to examine whether different frequencies of sleep fragmentation are associated with endpoint-specific alterations during colitis. We compared low- and high-frequency sleep fragmentation and evaluated clinical and histological colitis outcomes together with fecal microbiota composition, SCFA profiles, mucosal inflammatory mediators, colonic CD4^+^ T-cell subsets, and epithelial barrier-associated markers.

## 2 Materials and methods

### 2.1 Experimental animals

Male C57BL/6 mice (10 weeks old; 25–28 g) were purchased from the National Laboratory Animal Center (Taipei, Taiwan). A total of 40 mice were housed under controlled conditions (22–24 °C; 55–60% humidity) with a 12 h light/12 h dark cycle (lights on at 8:00 AM; lights off at 8:00 PM). Food and water were provided ad libitum. All procedures were approved by the Institutional Animal Care and Use Committee of Chang Gung Memorial Hospital (IACUC No. 2021120803).

### 2.2 Experimental design and groups

Forty mice were randomized into five groups (n = 8 per group):

1. Control: no intervention.
2. DSS: DSS-induced colitis only.
3. DSS + LSF: DSS-induced colitis with low-frequency sleep fragmentation.
4. DSS + HSF: DSS-induced colitis with high-frequency sleep fragmentation.
5. HSF: high-frequency sleep fragmentation only.

The study timeline and apparatus are shown in **Figure 1** and **Video S1**. Although eight mice were initially assigned to each group, the effective sample size varied across downstream assays because of sample availability and technical quality control. The actual sample size for each experiment is indicated in the corresponding figure legends.

**Figure 1:**
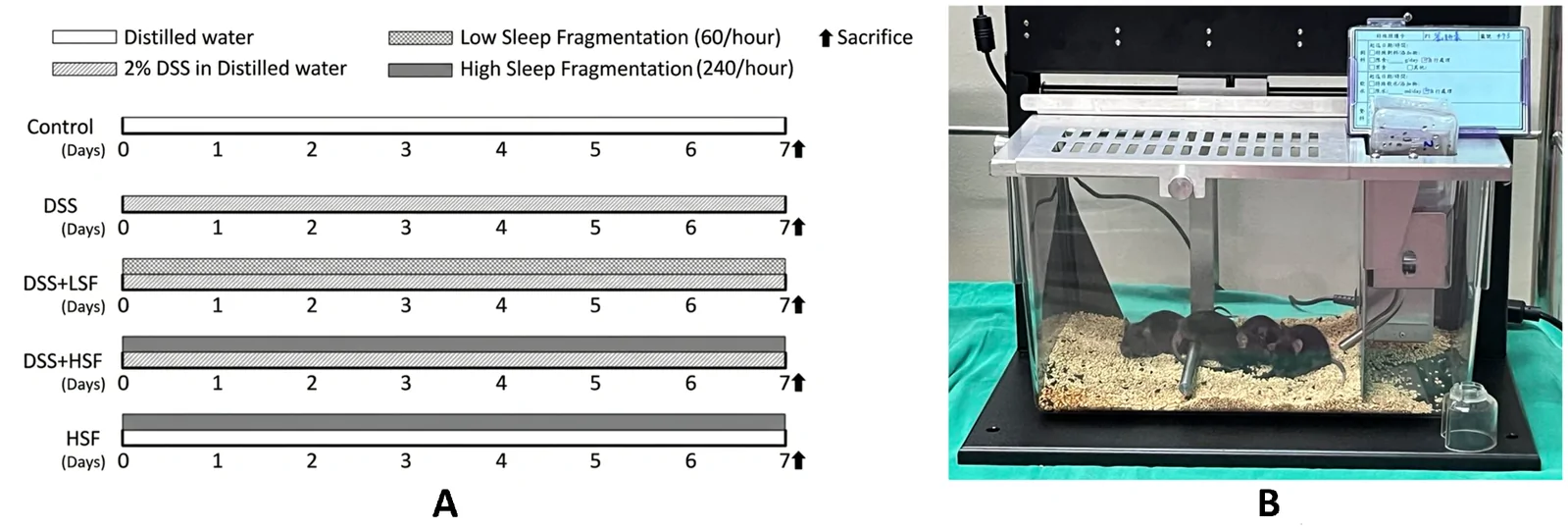
Experimental design and sleep fragmentation apparatus. **(A)** This study consisted of five groups: Control group, DSS group, DSS + LSF group, DSS + HSF group, and HSF group; **(B)** Sleep fragmentation apparatus.

### 2.3 Sleep fragmentation paradigm

Sleep fragmentation (SF) was induced using a motor-driven sweeping bar system (Lafayette Instrument Company, Lafayette, IN, USA; No. 80391) as previously described (20). The sweeping bar moved across the cage floor and was designed to interrupt sleep continuity. Two SF frequencies were applied during the light phase (08:00–20:00): LSF (one sweep/min; 60 sweeps/h) and HSF (four sweeps/min; 240 sweeps/h). These frequencies were selected *a priori* to provide a fourfold contrast in apparatus-defined interruption frequency while maintaining the same 12-h exposure window.

Sleep architecture was not directly measured by electroencephalography/electromyography (EEG/EMG) in the present study. Therefore, the terms LSF and HSF refer to apparatus-defined low- and high-frequency sleep-interruption protocols rather than objectively quantified differences in sleep architecture.

### 2.4 DSS-induced colitis model

Colitis was induced with 2% dextran sodium sulfate (DSS; molecular weight 36–50 kDa) in drinking water for 7 consecutive days (19, 21). A 2% DSS concentration was selected to induce a moderate acute colitis phenotype in C57BL/6 mice, allowing evaluation of the modifying effects of sleep fragmentation while minimizing excessive mortality. Water intake was measured daily, and both food and water were replenished every 2 days.

### 2.5 Sample collection and euthanasia

At the experimental endpoint on day 7, mice were deeply anesthetized with 4–5% isoflurane delivered in oxygen until loss of pedal withdrawal and corneal reflexes was confirmed. While under deep anesthesia, mice were euthanized by exsanguination via cardiac puncture, and death was confirmed by cessation of heartbeat and respiration before tissue harvesting.

### 2.6 Evaluation of experimental results

Colitis severity was evaluated by (1) clinical indicators; (2) macroscopic colon measurements; (3) histology and immunohistochemistry; (4) biochemical markers; (5) flow cytometry of colonic CD4^+^ T-cell subsets; and (6) fecal microbiota and short-chain fatty acids (SCFAs).

#### 2.6.1 Clinical indicators

Body weight and food intake were recorded daily at 8:00 AM. Stool consistency was observed and recorded (**Tables 1**, **2**). Fecal occult blood was assessed using a commercial colorimetric reagent (BaSO Enterprises, Taiwan) according to the manufacturer’s instructions. Bleeding severity was scored semi-quantitatively on a 0–4 scale based on the time to color development: 0, no reaction; 1, color development within 1–2 min; 2, within 1 min; 3, within 10 s; and 4, immediate color development. The Disease Activity Index (DAI) was calculated as the sum of the body weight loss score, stool consistency score, and fecal occult blood score, yielding a total score ranging from 0 to 12 (20).

**Table 1:** The scoring for weight change.

Weight loss percentage	score
1-5%	1
5-10%	2
10-20%	3
>20%	4

**Table 2:** The scoring for stool consistency.

Stool Pellet Description	Score
Normal stool pellets	0
Loose stool shape (paste-like but not adherent to the anus)	2
Watery stool (watery and adherent to the anus)	4

#### 2.6.2 Macroscopic evidence of the colon

On the final day, mice were euthanized and colons were excised. Colon length (from cecum to the anal verge) was measured as a macroscopic indicator of colitis severity. Fecal samples were collected for downstream microbiome/SCFA analyses.

#### 2.6.3 Microscopic assessment of the colon

Colon tissues were fixed in 4% formalin in phosphate-buffered saline, paraffin-embedded, sectioned at 7 μm, and stained with hematoxylin and eosin (H&E). An independent pathologist evaluated histopathology. Inflammation severity was scored using the criteria in **Table 3** (22, 23). The total histological score was calculated as the sum of six domains: goblet cell loss, mucosal thickening, inflammatory cell infiltration, submucosal cell infiltration, architectural destruction, and ulceration. Each domain was scored from 0 to 4 according to the extent of involvement, yielding a total score range of 0–24.

**Table 3:** Histological criteria for the degree of inflammation in the colon tissue.

Criteria	0	1	2	3	4
Goblet cell loss	0	<25%	25-50%	50-75%	75-100%
Mucosal thickening	0	<25%	25-50%	50-75%	75-100%
Inflammatory cells	0	<25%	25-50%	50-75%	75-100%
Submucosal cell infiltration	0	<25%	25-50%	50-75%	75-100%
Architectural destruction	0	<25%	25-50%	50-75%	75-100%
Ulcers	0	<25%	25-50%	50-75%	75-100%

Immunohistochemistry (IHC) was performed using an automated staining system (Bond-III, Leica Biosystems) with on-board deparaffinization and antigen retrieval (Bond Epitope Retrieval Solution 1). Sections were incubated with primary antibodies at room temperature for 30 min, followed by polymer HRP anti-mouse/rabbit IgG detection and DAB visualization, then counterstained with hematoxylin, dehydrated, cleared, and mounted. Staining was quantified at 200× magnification by measuring the percentage of positive-stained area using ImageJ. Primary antibodies and dilutions were:

- TJP1 (rabbit polyclonal, 1:200; Boster)
- OCLN (rabbit polyclonal, 1:150; CUSABIO)
- AQP4 (rabbit polyclonal, 1:200; Abclonal)

#### 2.6.4 Biochemical markers

Blood samples were collected via intracardiac puncture under isoflurane anesthesia and processed to obtain plasma. The plasma was subsequently diluted 1:10, 000, and C-reactive protein (CRP) levels were quantified using the Mouse C-Reactive Protein/CRP Quantikine ELISA Kit (MCRP00; R&D Systems, Minneapolis, MN, USA). Plasma corticosterone concentrations were measured using a commercially available competitive ELISA kit (EIACORT, Thermo Fisher Scientific, MA, USA) according to the manufacturer’s instructions.

For colonic cytokine profiling, colon tissues were homogenized in 700 μL of Tris-HCl buffer supplemented with protease inhibitors (Sigma). The homogenates were centrifuged at 13, 000 × g for 20 min at 4 °C, and the resulting supernatants were collected and stored at −80 °C. Cytokine concentrations were evaluated using the Bio-Plex Pro Mouse Cytokine 23-Plex Immunoassay (Bio-Rad) according to the manufacturer’s protocol. This multiplex panel assessed the following 23 cytokines and chemokines: IL-1α, IL-1β, IL-2, IL-3, IL-4, IL-5, IL-6, IL-9, IL-10, IL-12(p40), IL-12(p70), IL-13, IL-17A, Eotaxin, G-CSF, GM-CSF, IFN-γ, KC, MCP-1 (MCAF), MIP-1α, MIP-1β, RANTES, and TNF-α.

To assess colonic myeloperoxidase (MPO) levels as an indicator of neutrophil infiltration, minced colon tissues were first washed with 500 μL of PBS (centrifuged at 2, 000 rpm for 5 min, discarding the supernatant). The tissue pellets were then lysed in 250 μL of RIPA buffer, sonicated for 10 cycles (2 s on, 1 s off), and centrifuged at 14, 000 rpm for 15 min at 4 °C. The supernatants were harvested for MPO quantification via ELISA and concurrent total protein determination. Final MPO levels were normalized to the total protein concentration and expressed as pg of MPO per mg of protein (pg/mg).

#### 2.6.5 Flow cytometry for colonic CD4^+^ T-cell subsets

Lamina propria mononuclear cells were isolated from colon tissue based on established protocols with minor modifications (24). Colons were flushed with PBS, opened longitudinally, cut into 1 cm segments, and washed twice in PBS containing 3 mmol/L EDTA (10 min at 37 °C, rotation). Tissues were then washed twice in RPMI-1640 containing 1% FCS, 1 mM EGTA, and 1.5 mmol/L MgCl_2_ (15 min at 37 °C, rotation), rinsed with PBS, minced, and digested in RPMI-1640 containing 20% FCS and collagenase IV (100 U/mL; Merck) for 60 min at 37 °C with rotation. Digests were filtered through a 40-μm strainer and washed in RPMI-1640.

Cells were stained with a fixable viability dye (eFluor 780) and surface markers, followed by fixation/permeabilization for intracellular staining. CD4^+^ T-cell subsets were quantified within the CD4^+^ gate as Th1 (IFN-γ^+^), Th2 (IL-13^+^), Th17 (IL-17A^+^), and Treg (Foxp3^+^). Acquisition was performed on an LSR II flow cytometer using DIVA software.

**Table 4** outlines the markers, fluorochromes, and staining methods used to identify and quantify colonic CD4^+^ T-cell subsets.

**Table 4:** Flow cytometry staining panel for colonic CD4^+^ T-cell subset analysis.

Marker	Fluorochrome	Staining	Dilution
Viability dye	eFluor 780	Surface	per manufacturer
CD4	PerCP/Cy5.5	Surface	1:300
IFN-γ	APC	Intracellular	1:50
IL-13	PE/Cy7	Intracellular	1:200
IL-17a	PE	Intracellular	1:50
Foxp3	BV421	Intracellular	1:50

#### 2.6.6 Fecal microbiota and short-chain fatty acids

Fecal samples were collected from opened colons at sacrifice and stored at −80 °C. DNA extraction and 16S rRNA sequencing were performed by BIOTOOLS CO. LTD. (Taiwan). Sequencing data were demultiplexed and imported into QIIME2 (v2022.11) (25). Primers were trimmed using cutadapt (forward: CCTACGGGNGGCWG; reverse: GACTACHVGGGTATCTAATCC). Denoising and ASV inference were performed using DADA2 in QIIME2 (26), with paired-end trimming (PE300 V3–V4; forward 280 bp, reverse 220 bp) and MaxEE thresholds (forward 2, reverse 2). Paired reads were merged (minimum overlap 12 bp; no mismatches) and chimeras were removed.

Taxonomy was assigned using the QIIME2 feature-classifier (classify-sklearn) (27). Multiple sequence alignment was performed using MAFFT (28), and a phylogenetic tree was constructed using FastTree2 (29). Sequence depth was normalized to the minimum reads across samples for downstream diversity analyses.

Alpha diversity indices and beta diversity were computed in QIIME2. Beta diversity was evaluated using weighted UniFrac distances (30, 31), and visualized using ordination methods as appropriate.

To identify taxa with differential abundance between groups while accounting for sparse count structure, metagenomeSeq was applied with multiple testing correction (FDR) (32). In addition, LEfSe was used to identify discriminative microbial biomarkers with an LDA score cutoff of 4.0, and results were visualized using cladograms and LDA bar plots (33).

SCFA extraction and GC–MS. SCFAs were extracted from 25 mg feces with 500 μL 10% isobutanol, homogenized (6, 000 rpm, 20 s × 2; 30 s interval), and centrifuged at 12, 000 rpm for 30 min. Derivatization was performed using NaOH/chloroform partitioning and isobutyl chloroformate chemistry, followed by hexane extraction. SCFAs were analyzed on a Bruker 436-GC coupled to a Bruker EVOQ mass spectrometer using MSWS 8.2 software, a VF-5ms 30 m × 0.25 mm × 0.5 μm column, helium carrier (1.0 mL/min), and an oven program of 40 °C (5 min) to 310 °C (10 °C/min). Injection volume was 2.0 μL with 10:1 split; inlet/transfer line/ion source temperatures were 260/280/250 °C; electron energy 70 eV.

### 2.7 Statistical analysis

Data are presented as mean ± standard error of the mean (SEM) unless otherwise indicated. Given the small sample sizes and without assuming normality, non-parametric analyses were used for endpoint comparisons. For each endpoint, differences among the five experimental groups were first assessed using the Kruskal-Wallis test. When *post hoc* comparisons were performed, Dunn’s multiple-comparison test was used for pre-specified pairwise comparisons focused on the DSS-treated groups: DSS vs. DSS+LSF, DSS vs. DSS+HSF, and DSS+LSF vs. DSS+HSF. Adjusted p-values< 0.05 were considered statistically significant. For longitudinal body weight and DAI data, daily curves were shown to illustrate temporal trends. Formal group-level comparisons were performed using day-7 body weight change and DAI area under the curve (AUC), respectively.

Microbiome alpha-diversity indices were analyzed using the Kruskal-Wallis test followed by Dunn’s correction. Beta diversity was analyzed using weighted UniFrac-based ordination and microbiome-specific statistical methods. Differentially abundant taxa were assessed using metagenomeSeq with false discovery rate correction, and LEfSe was used as an exploratory biomarker-discovery approach. Statistical analyses were performed using IBM^®^ SPSS^®^ Statistics Version 22.0 and GraphPad Prism 10.

## 3 Results

### 3.1 Clinical and macroscopic indices showed group-level variation during DSS-induced colitis

To evaluate clinical disease severity, daily body weight change, disease activity index (DAI), and endpoint colon length were assessed (**Figures 2A–C**). Daily food and water intake are presented in **Supplementary Figures S1**, **S2**.

**Figure 2:**
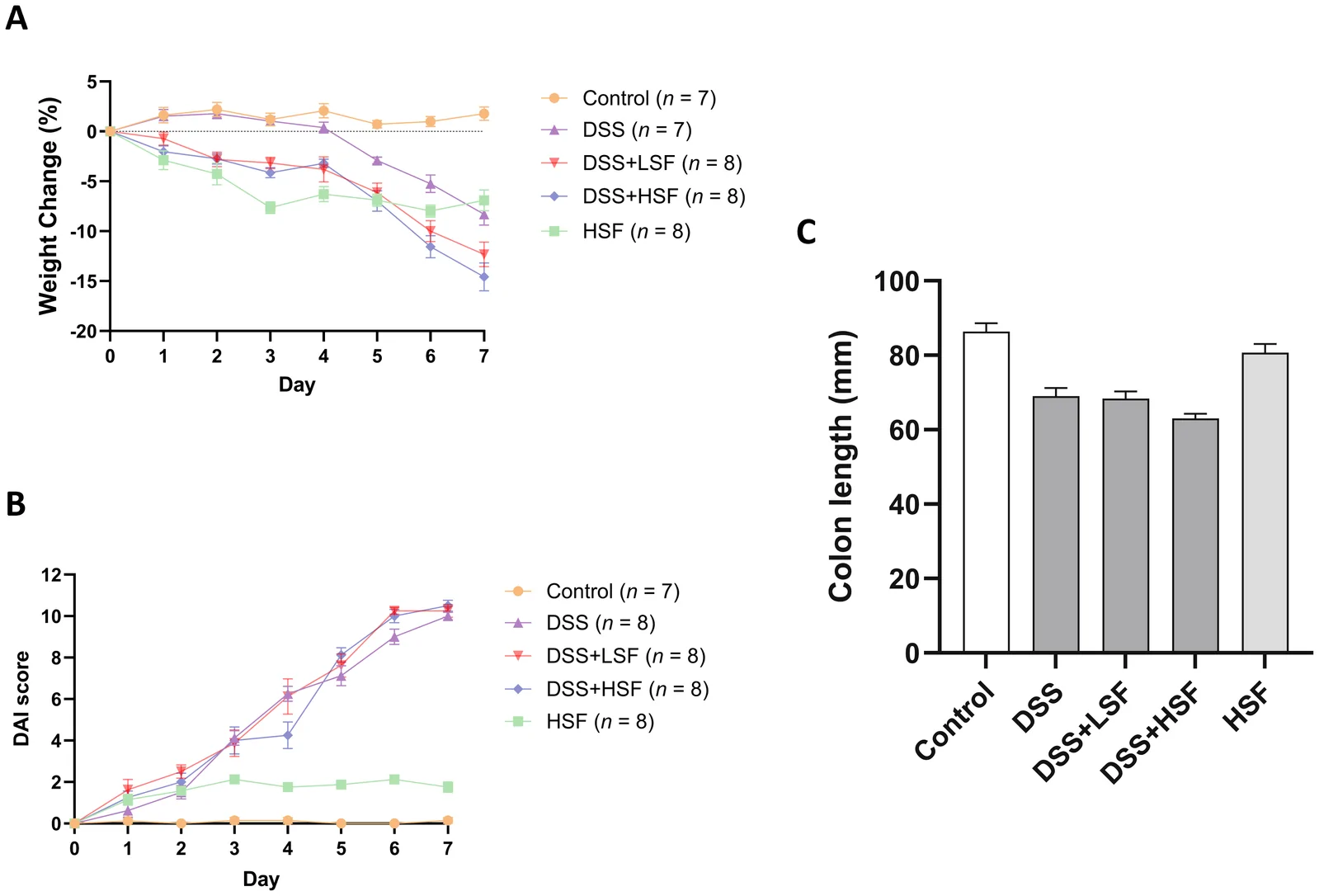
Physiological metrics of mice. **(A)** Body weight change (%) (Control, *n* = 7; DSS, *n* = 7; DSS+LSF, *n* = 8; DSS+HSF, *n* = 8; HSF, *n* = 8). **(B)** Daily DAI score (Control, *n* = 7; DSS, *n* = 8; DSS+LSF, *n* = 8; DSS+HSF, *n* = 8; HSF, *n* = 8). **(C)** Colon length (Control, *n* = 6; DSS, *n* = 8; DSS+LSF, *n* = 6; DSS+HSF, *n* = 6; HSF, *n* = 7). Kruskal-Wallis tests showed significant overall group differences for day-7 body weight change, DAI AUC, and colon length. Pre-specified Dunn’s *post hoc* comparisons among DSS, DSS+LSF, and DSS+HSF were not significant.

Body weight was monitored longitudinally throughout the 7-day experimental period. DSS-treated mice showed progressive body weight loss, consistent with acute DSS-induced colitis. Day-7 body weight change differed significantly among the five experimental groups by Kruskal-Wallis test (p< 0.0001). However, the pre-specified Dunn’s *post hoc* comparisons among DSS, DSS+LSF, and DSS+HSF did not reach statistical significance.

DAI increased over time in DSS-treated mice and showed variation across experimental groups. To avoid overinterpretation of multiple daily pairwise comparisons, the daily DAI curve was used to illustrate temporal progression, whereas overall disease burden was summarized using DAI area under the curve (AUC). DAI AUC differed significantly among the five experimental groups by Kruskal-Wallis test (p< 0.0001), but the pre-specified Dunn’s *post hoc* comparisons among DSS-treated groups were not statistically significant.

Endpoint colon length also differed significantly among the five experimental groups by Kruskal-Wallis test (p = 0.0001). Descriptively, colon shortening appeared more pronounced in DSS+HSF than in the other DSS-treated groups. However, corrected *post hoc* comparisons among DSS-treated groups did not reach statistical significance.

### 3.2 DSS+LSF showed the most prominent descriptive histological injury profile among DSS-treated groups

Colonic injury was evaluated by H&E staining and histological scoring (**Figures 3A–F**). Control and HSF-only groups showed relatively preserved crypt architecture and goblet cell morphology, with minimal inflammatory infiltration. In contrast, DSS-treated groups showed epithelial injury characterized by goblet cell depletion, inflammatory cell infiltration, mucosal thickening, and architectural disruption.

**Figure 3:**
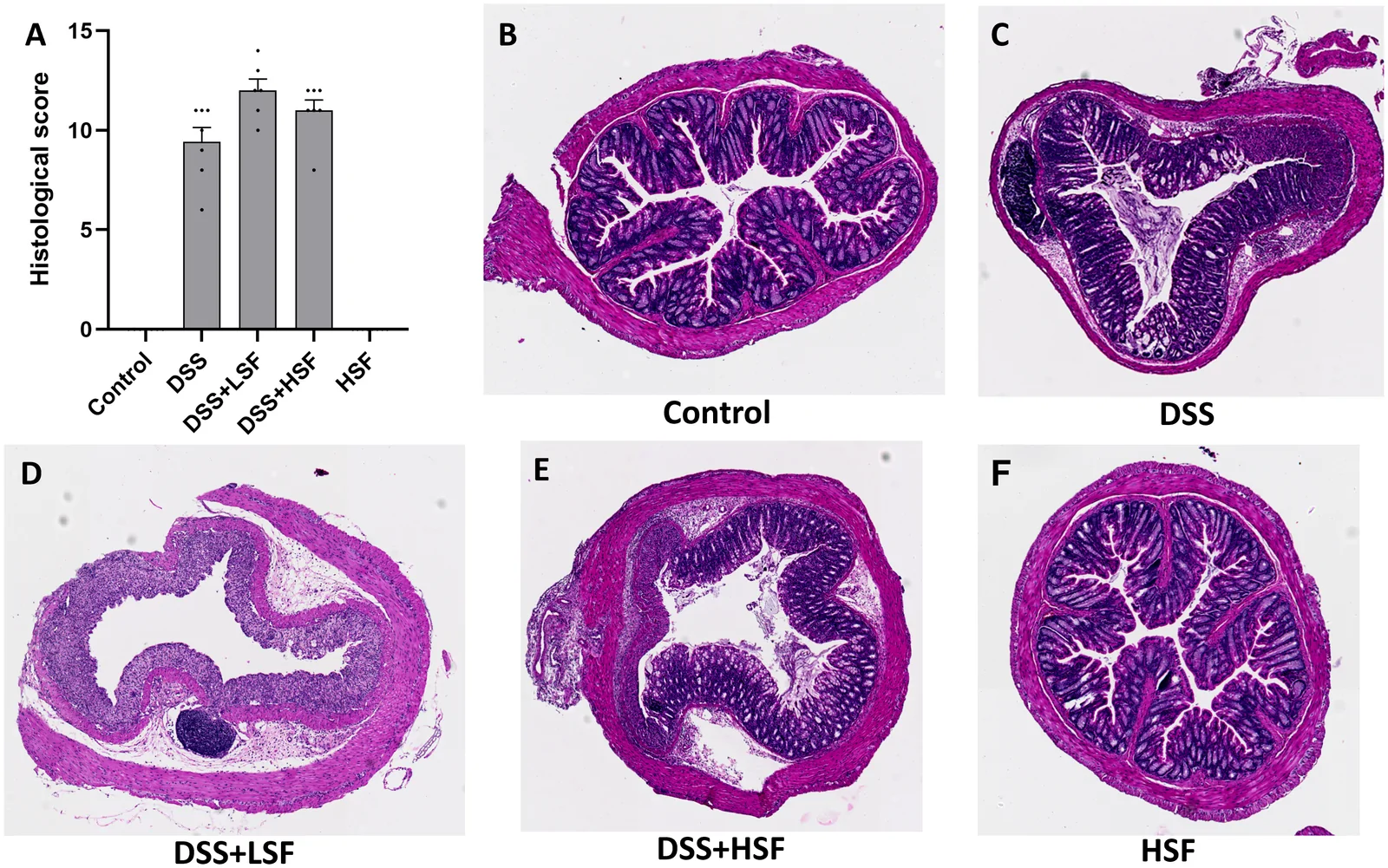
Histological inflammation and representative H&E-stained colon sections. **(A)** Quantified histological inflammation scores in each group. **(B–F)** Representative colon sections stained with hematoxylin and eosin (H&E) at ×40 magnification. (Control, *n* = 7; DSS, *n* = 7; DSS+LSF, *n* = 6; DSS+HSF, *n* = 7; HSF, *n* = 8).

Among the DSS-treated groups, DSS+LSF displayed the highest descriptive histological injury score and appeared to show more prominent mucosal inflammatory damage in the representative H&E sections. However, the pre-specified pairwise comparisons among DSS, DSS+LSF, and DSS+HSF did not remain statistically significant after Dunn’s multiple-comparison correction.

### 3.3 DSS+HSF showed a descriptive reduction in barrier-associated protein expression

To assess epithelial barrier-associated markers, OCLN, TJP-1, and AQP4 were quantified by immunohistochemistry (**Figure 4**). Across DSS-treated groups, barrier-associated protein staining showed descriptive variation. DSS+HSF appeared to show lower OCLN and TJP-1 staining than the other DSS-treated groups. AQP4 staining also varied across groups, but without a clear frequency-specific pattern. However, after Dunn’s multiple-comparison correction, the pre-specified pairwise comparisons among DSS, DSS+LSF, and DSS+HSF did not reach statistical significance for OCLN, TJP-1, or AQP4.

**Figure 4:**
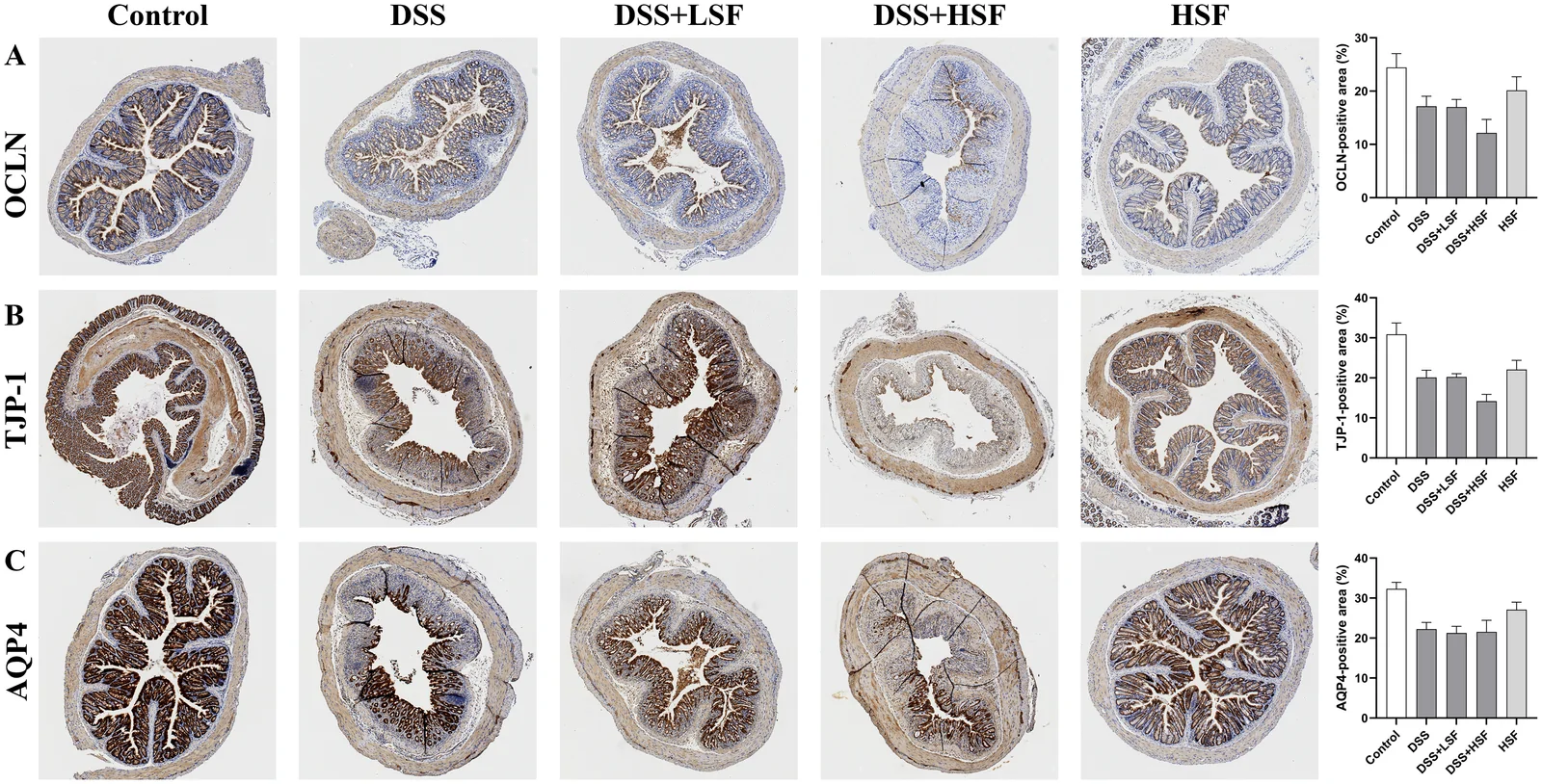
Immunohistochemical assessment of intestinal barrier–associated proteins. Representative immunohistochemical staining of **(A)** OCLN, **(B)** TJP-1, and **(C)** AQP4 in colonic sections. Images were acquired at ×40 magnification. (Control, *n* = 6; DSS, *n* = 6; DSS+LSF, *n* = 6; DSS+HSF, *n* = 6; HSF, *n* = 6).

### 3.4 Sleep fragmentation increased corticosterone during DSS colitis, while MPO differed between fragmentation frequencies

Stress-related and inflammatory markers were assessed using plasma corticosterone, CRP, colonic cytokine/chemokine profiling, and tissue MPO levels (**Figure 5**). Plasma corticosterone concentrations were significantly higher in DSS+LSF than in DSS alone (adjusted p = 0.0062) and in DSS+HSF than in DSS alone (adjusted p = 0.0059). Corticosterone levels did not differ significantly between DSS+LSF and DSS+HSF.

**Figure 5:**
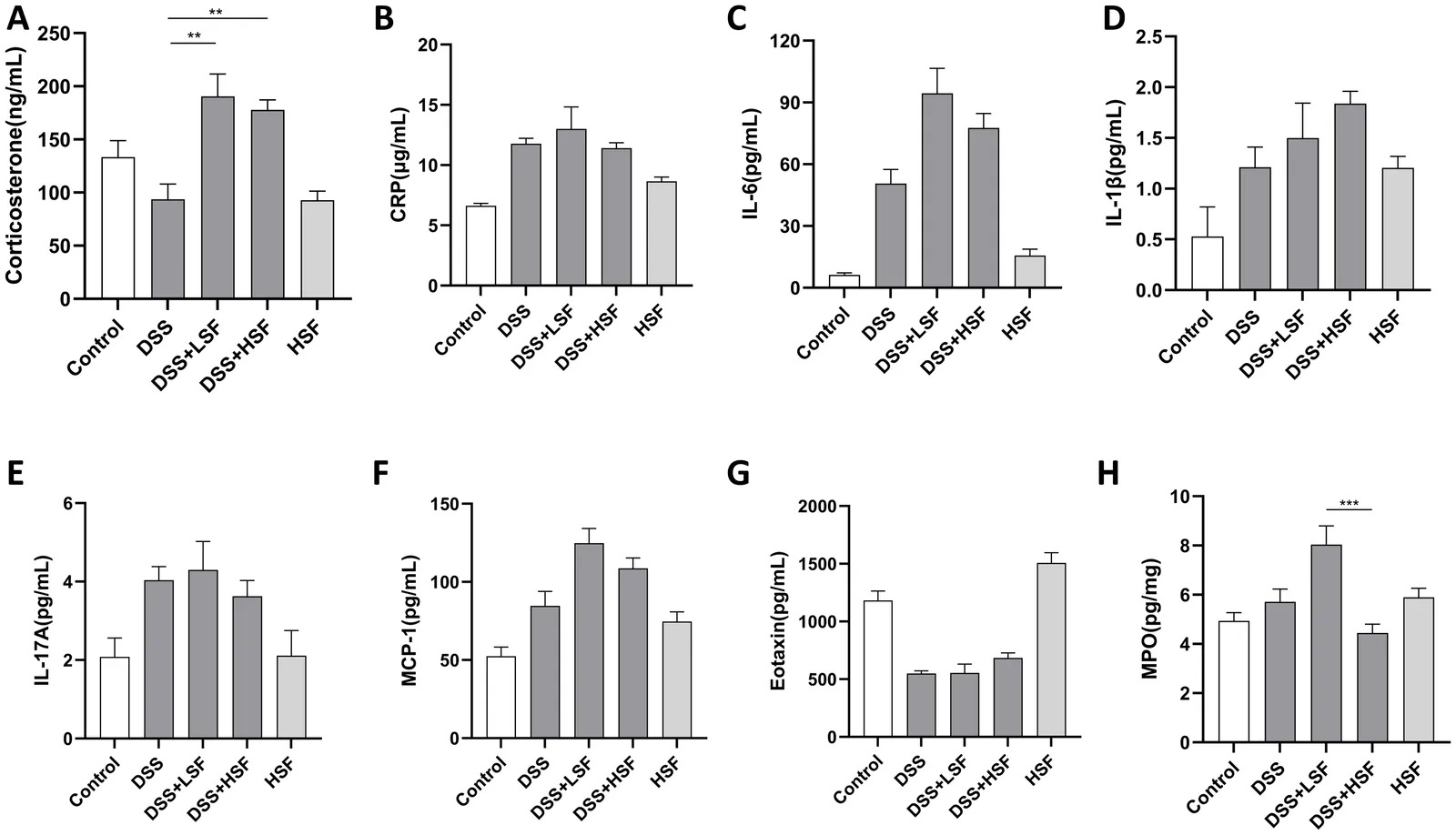
Stress-related and inflammatory markers across experimental groups. **(A)** Plasma corticosterone (Control, *n* = 6; DSS, *n* = 7; DSS+LSF, *n* = 6; DSS+HSF, *n* = 8; HSF, *n* = 8) **(B)** CRP (Control, *n* = 7; DSS, *n* = 6; DSS+LSF, *n* = 6; DSS+HSF, *n* = 7; HSF, *n* = 7) **(C)** IL-6 (Control, *n* = 7; DSS, *n* = 6; DSS+LSF, *n* = 6; DSS+HSF, *n* = 7; HSF, *n* = 6) **(D)** IL-1β (Control, *n* = 4; DSS, *n* = 6; DSS+LSF, *n* = 5; DSS+HSF, *n* = 6; HSF, *n* = 7) **(E)** IL-17 (Control, *n* = 6; DSS, *n* = 7; DSS+LSF, *n* = 4; DSS+HSF, *n* = 8; HSF, *n* = 7) **(F)** MCP-1 (Control, *n* = 5; DSS, *n* = 7; DSS+LSF, *n* = 5; DSS+HSF, *n* = 6; HSF, *n* = 7) **(G)** Eotaxin (Control, *n* = 7; DSS, *n* = 8; DSS+LSF, *n* = 6; DSS+HSF, *n* = 6; HSF, *n* = 7) **(H)** MPO (Control, *n* = 5; DSS, *n* = 8; DSS+LSF, *n* = 6; DSS+HSF, *n* = 8; HSF, *n* = 7). **p< 0.01; ***p< 0.001 by Dunn’s multiple-comparison correction for pre-specified comparisons.

Among colonic inflammatory mediators, DSS with sleep fragmentation showed descriptive increases in selected cytokine and chemokine markers, including IL-6 and MCP-1, compared with DSS alone. However, after Dunn’s multiple-comparison correction, the pre-specified pairwise comparisons among DSS, DSS+LSF, and DSS+HSF did not reach statistical significance for CRP, IL-6, IL-1β, IL-17A, MCP-1, or eotaxin. MPO was the only inflammatory marker that retained a significant corrected difference between sleep-fragmentation frequencies, with higher levels in DSS+LSF than in DSS+HSF (adjusted p = 0.0009).

### 3.5 High-frequency sleep fragmentation was associated with increased colonic Th2 proportions during DSS colitis

Flow cytometry was used to evaluate colonic CD4^+^ T-cell subset distributions, including Th1, Th2, Th17, and Treg cells (**Figure 6**). Among the analyzed CD4^+^ T-cell subsets, Th2 was the only subset that retained significant differences among DSS-treated groups after Dunn’s multiple-comparison correction. DSS+HSF showed significantly higher Th2 proportions than DSS alone and DSS+LSF, whereas DSS and DSS+LSF did not differ significantly.

**Figure 6:**
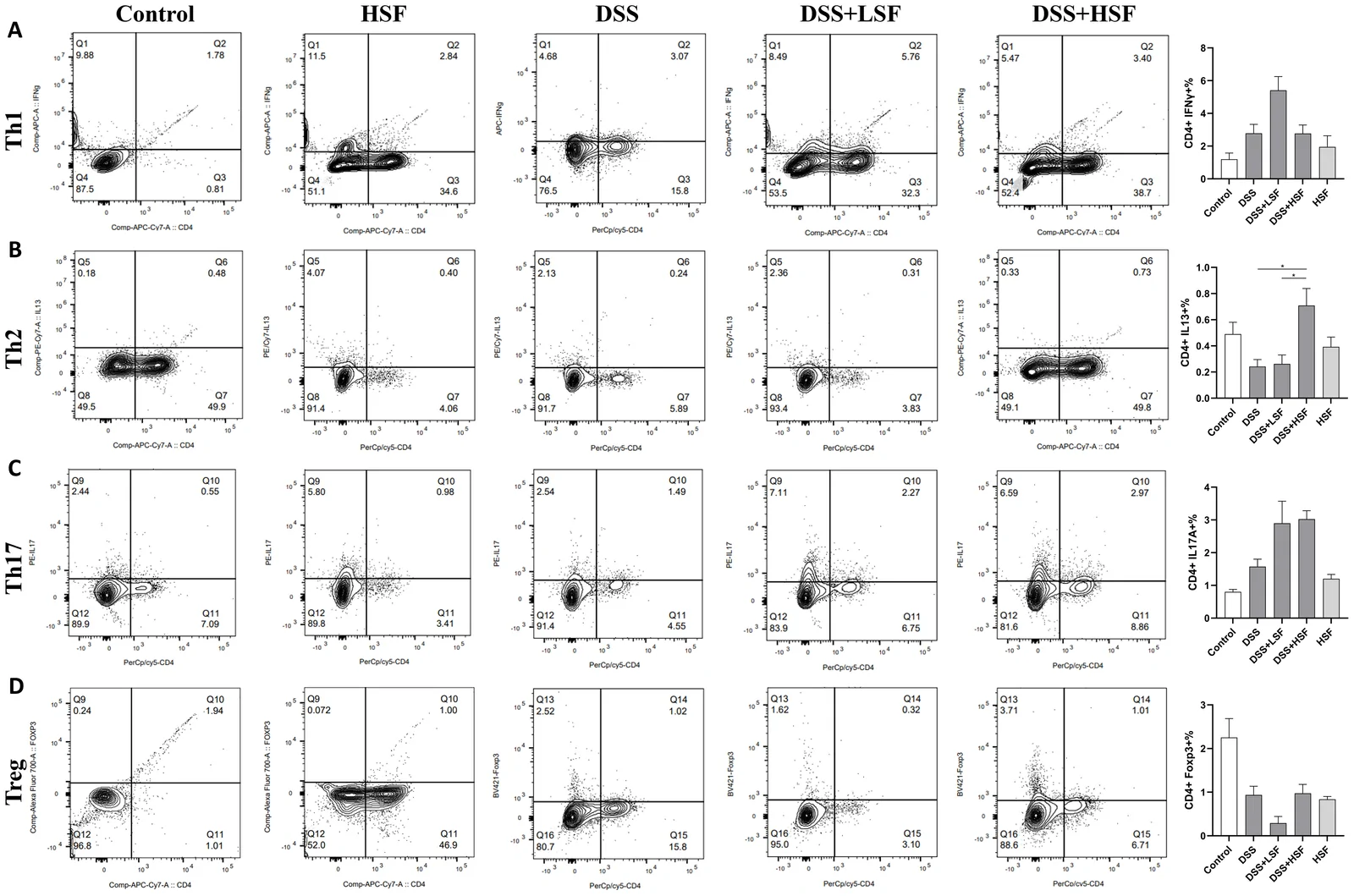
Flow cytometric profiling of colonic CD4+ T-cell subsets. Representative flow cytometry plots and quantification of **(A)** Th1 (CD4+IFN-γ+), **(B)** Th2 (CD4+IL-13+), **(C)** Th17 (CD4+IL-17A+), and **(D)** Treg (CD4+Foxp3+) cells across experimental groups. *p< 0.05 by Dunn’s multiple-comparison correction for Th2 comparisons. Other pre-specified comparisons among DSS-treated groups were not significant. CD4, cluster of differentiation 4; IFN-γ, interferon gamma; IL, interleukin; Foxp3, forkhead box P3.

Th1, Th17, and Treg cells also showed descriptive variation across DSS-treated groups. In particular, Treg proportions showed numerical separation between DSS+LSF and DSS+HSF, but this comparison did not reach statistical significance after Dunn’s multiple-comparison correction. The pre-specified corrected comparisons among DSS-treated groups were also not significant for Th1 or Th17 cells.

Together with the MPO finding, these results indicate that immune-related endpoints differed by fragmentation frequency, with MPO more prominent in DSS+LSF and Th2 proportions more prominent in DSS+HSF.

### 3.6 DSS+HSF showed a distinct weighted UniFrac distance profile during DSS colitis

Fecal microbiota were profiled using ASVs (**Figure 7**). Rarefaction curves approached saturation across groups, supporting adequate sequencing depth for downstream analyses. Shannon diversity and species richness showed descriptive variation across groups, with DSS+HSF appearing numerically higher than DSS+LSF in both alpha-diversity indices. However, the pre-specified Dunn’s *post hoc* comparisons among DSS, DSS+LSF, and DSS+HSF did not reach statistical significance for either Shannon diversity or species richness. For the weighted UniFrac distance metric, DSS+HSF showed significantly higher values than DSS alone (p = 0.0064). DSS vs. DSS+LSF and DSS+LSF vs. DSS+HSF did not reach statistical significance.

**Figure 7:**
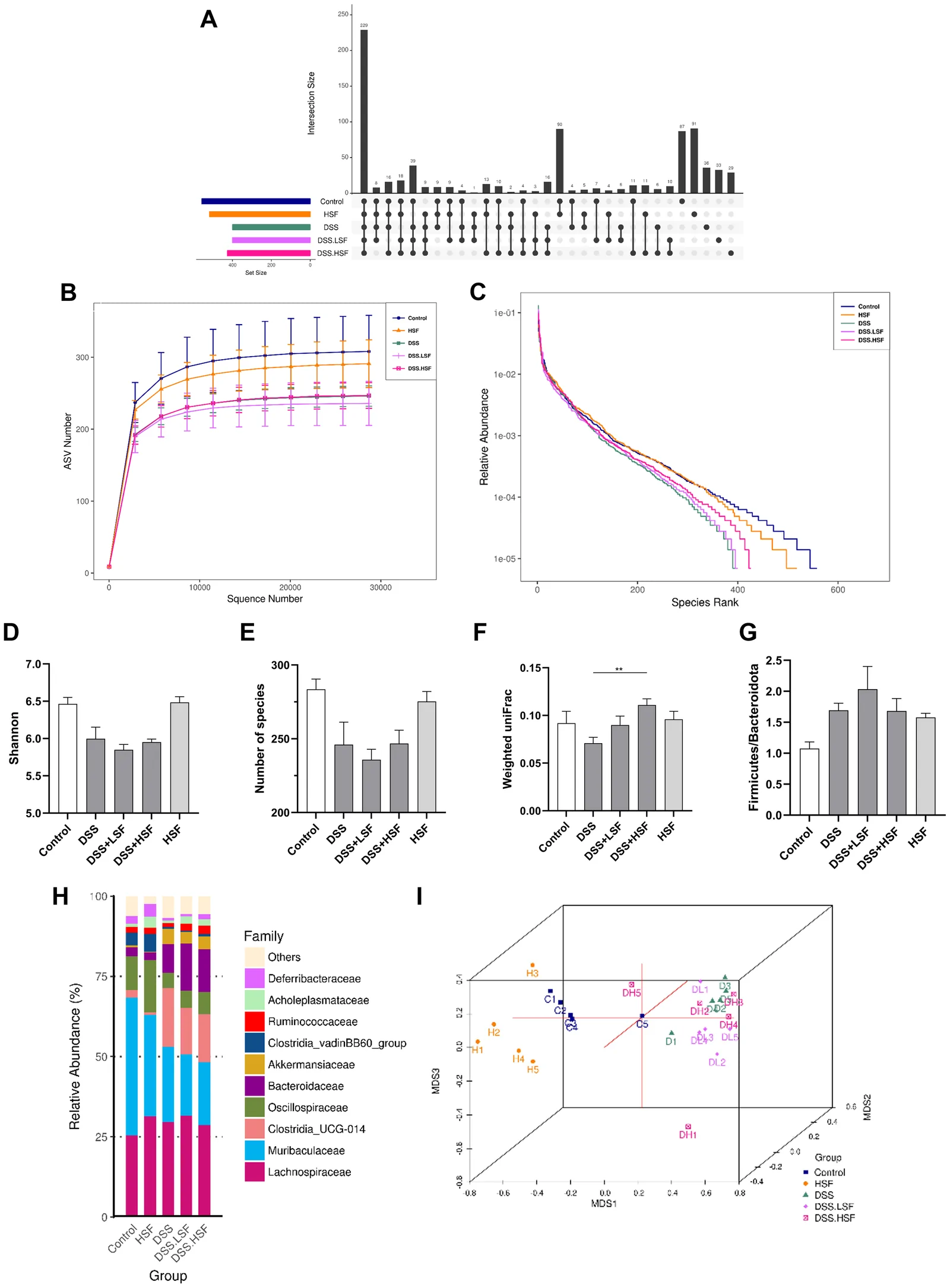
Gut microbiota profiling across experimental groups. **(A)** UpSet plot of shared and unique ASVs among groups. **(B)** Rarefaction curves. **(C)** Rank-abundance curves. **(D)** Shannon diversity index. **(E)** Species richness (observed ASVs). **(F)** Weighted UniFrac distance. **(G)** Firmicutes/Bacteroidota ratio. **(H)** Relative abundance of bacterial families. **(I)** Three-dimensional ordination based on weighted UniFrac distances. Data are mean ± SEM. **p< 0.01. ASV, amplicon sequence variant. (*n* = 4–5 in each group).

Taxonomic biomarker analyses further showed group-associated microbial signatures (**Figure 8**). Control samples were enriched for Muribaculaceae and related Bacteroidota lineages, whereas DSS samples were enriched for Verrucomicrobiota-related features, including Akkermansiaceae/Akkermansia. DSS+LSF was associated with enrichment of Bacteroidaceae/Bacteroides and Lachnospiraceae-related taxa. DSS+HSF showed fewer discriminative taxa, with Clostridales bacterium among the notable features. In the HSF-only group, enrichment was observed for Oscillospirales/Oscillospiraceae, Deferribacteraceae/Mucispirillum, and Clostridia vadinBB60-related taxa. Together, the alpha-diversity trends, weighted UniFrac distance metric, and taxonomic signatures showed that microbial community profiles differed between DSS+LSF and DSS+HSF.

**Figure 8:**
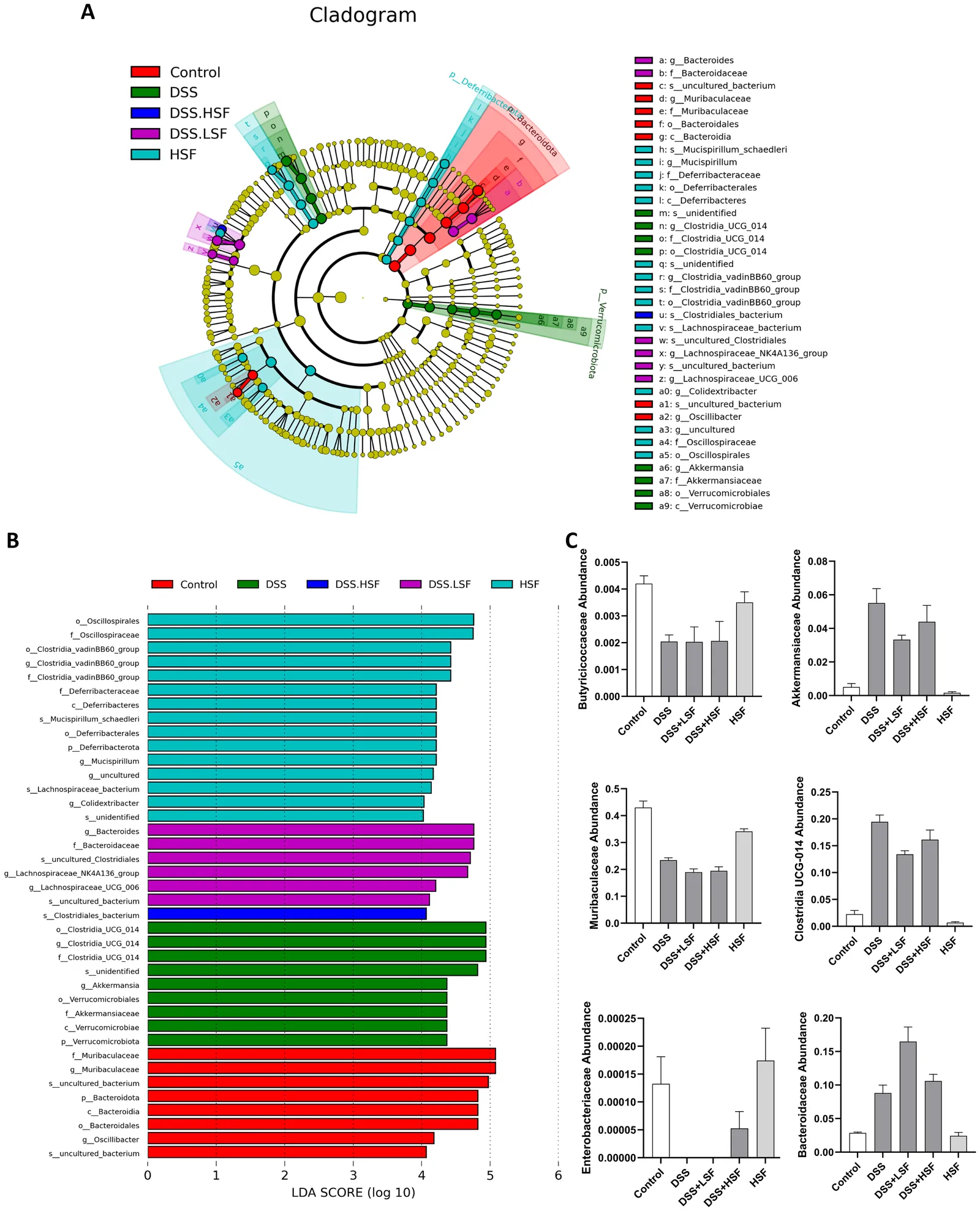
Differentially abundant taxa across experimental groups. **(A)** LEfSe cladogram showing taxonomic biomarkers enriched in each group. Concentric rings represent taxonomic ranks from phylum to genus/species, and node size reflects relative abundance; taxa without significant differences are shown in yellow, whereas significant biomarkers are colored by the group in which they are enriched. **(B)** LEfSe LDA score histogram (log10). Only taxa with LDA scores ≥ 4.0 are displayed. **(C)** Differential abundance results generated using metagenomeSeq, shown as relative abundance of representative significant taxa across groups. Data are mean ± SEM. LEfSe, linear discriminant analysis effect size; LDA, linear discriminant analysis. (*n* = 4–5 in each group).

### 3.7 Fecal SCFA profiles showed descriptive differences across DSS and sleep-fragmentation conditions

Fecal SCFA profiles are shown in **Figure 9**. DSS exposure was associated with broad descriptive reductions across several measured SCFAs. Compared with DSS alone, DSS+LSF showed numerically lower levels of major SCFAs, including acetate and propionate. DSS+HSF showed relatively higher levels of several SCFAs than DSS+LSF, including formate, acetate, propionate, pentanoate, and 3-methylbutyrate. In the HSF-only group, acetate and propionate were numerically reduced, whereas formate and butyrate appeared higher. These SCFA findings were described as endpoint metabolite patterns.

**Figure 9:**
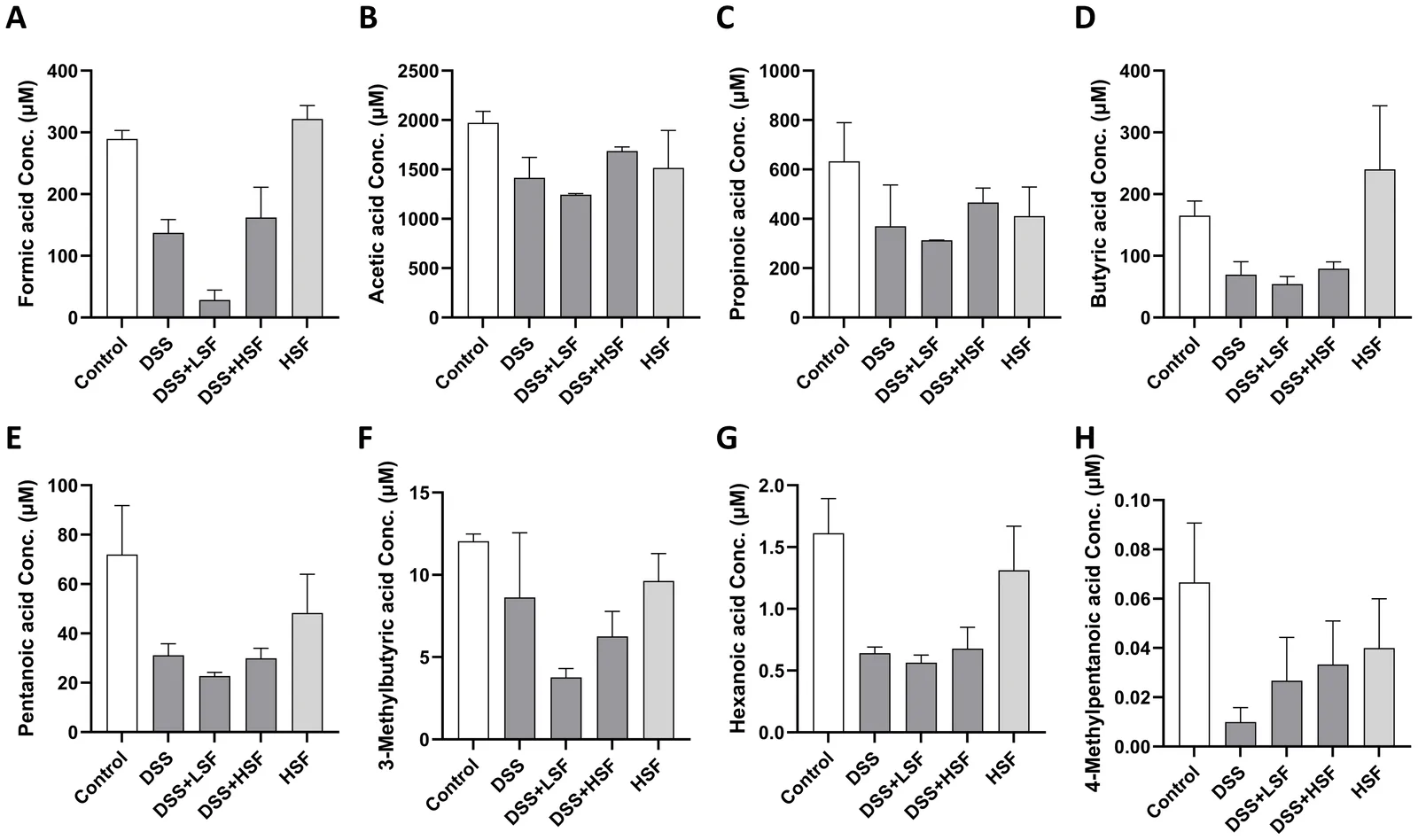
Fecal short-chain fatty acid concentrations across experimental groups. **(A)** Formate, **(B)** acetate, **(C)** propionate, **(D)** butyrate, **(E)** pentanoate, **(F)** 3-methylbutyrate, **(G)** hexanoate, and **(H)** 4-methylpentanoate concentrations (µM) are shown for each group. Data are mean ± SEM. (*n* = 4–5 in each group).

## 4 Discussion

### 4.1 Principal findings

In this study, we examined whether low- and high-frequency sleep fragmentation were associated with distinct endpoint profiles in DSS-induced colitis. Overall, sleep fragmentation did not produce a simple linear worsening pattern across all measured outcomes. Instead, different clinical, inflammatory, immune, microbial, metabolic, and barrier-associated endpoints showed different patterns across the DSS+LSF and DSS+HSF groups.

Among the pre-specified corrected comparisons within DSS-treated groups, plasma corticosterone was increased in both DSS+LSF and DSS+HSF compared with DSS alone, MPO levels were higher in DSS+LSF than in DSS+HSF, colonic Th2 proportions were higher in DSS+HSF than in DSS and DSS+LSF, and the weighted UniFrac distance metric was higher in DSS+HSF than in DSS alone. Other findings were descriptive: DSS+LSF showed the highest histological injury score and numerical increases in selected inflammatory mediators, whereas DSS+HSF showed lower OCLN and TJP-1 staining. Fecal SCFA profiles also differed descriptively across fragmentation conditions. Together, these findings indicate that different biological domains may respond differently to sleep-fragmentation frequency rather than following a uniform severity gradient.

### 4.2 Sleep fragmentation, stress response, and clinical phenotype during DSS colitis

Sleep disturbance is clinically relevant in IBD and has been associated with disease activity and subsequent relapse risk in longitudinal observations (2, 5, 6). Experimental evidence also supports the biological plausibility that sleep disruption can aggravate intestinal inflammation, as sleep deprivation has been shown to worsen inflammation and delay recovery in DSS-induced colitis (18). Sleep fragmentation represents a distinct form of sleep disruption (15), and experimental studies have shown that acute fragmentation can activate the hypothalamic–pituitary–adrenal response, increase corticosterone, and produce tissue- and time-dependent inflammatory changes (34, 35).

In the present study, corticosterone was significantly higher in both DSS+LSF and DSS+HSF than in DSS alone, but did not differ between the two fragmentation frequencies. Thus, both protocols were associated with stress-response activation, without evidence of a simple frequency-dependent increase in corticosterone. Clinical and macroscopic indices also showed significant overall differences across the five experimental groups; however, corrected comparisons among DSS, DSS+LSF, and DSS+HSF were not significant. These findings therefore support overall variation across experimental conditions rather than definitive evidence that one fragmentation frequency uniformly aggravated clinical disease severity.

### 4.3 Histological injury and barrier-associated responses showed non-parallel patterns

Histological and barrier-associated endpoints did not change in parallel across DSS-treated groups. DSS+LSF showed the highest descriptive histological injury score, whereas DSS+HSF showed lower OCLN and TJP-1 staining. However, none of these pairwise differences remained statistically significant after Dunn’s multiple-comparison correction. This divergence suggests that mucosal structural injury and barrier-associated protein expression may represent partially distinct response domains rather than a single graded continuum of disease severity.

Epithelial barrier dysfunction is a key feature of intestinal inflammation, and disruption of tight-junction proteins such as OCLN and TJP-1 may alter epithelial permeability and mucosal responses to luminal stimuli (12, 36, 37). Experimental studies have also shown that sleep deprivation can impair intestinal barrier integrity, reduce goblet-cell and tight-junction-associated markers, and promote mucosal injury (38, 39). In addition, disruption of colonic epithelial circadian rhythms has been reported to worsen DSS-induced colitis and increase intestinal permeability (13). These findings provide biological context for the descriptive barrier-associated changes observed in DSS+HSF. Nevertheless, because corrected comparisons for OCLN, TJP-1, and AQP4 were not significant, the present findings should not be interpreted as definitive evidence that HSF causes greater barrier impairment than LSF. Rather, the non-parallel patterns of histological injury and barrier-associated markers further support an endpoint-specific response to different sleep-fragmentation frequencies.

### 4.4 Different fragmentation frequencies were associated with distinct immune response patterns

Immune-related endpoints showed different patterns across sleep-fragmentation frequencies. Among inflammatory mediators, DSS+LSF showed descriptive increases in IL-6 and MCP-1, although these differences did not remain significant after multiple-comparison correction. In contrast, MPO was significantly higher in DSS+LSF than in DSS+HSF. Among colonic CD4^+^ T-cell subsets, Th2 was the only subset that retained significant corrected differences, with higher proportions in DSS+HSF than in DSS and DSS+LSF. Th1, Th17, and Treg showed descriptive variation but no significant corrected differences among DSS-treated groups.

These findings indicate that sleep fragmentation was not associated with a uniform amplification of mucosal inflammation. Rather, neutrophil-associated inflammatory activity, reflected by MPO, was more prominent in DSS+LSF, whereas a selective Th2-associated response was more evident in DSS+HSF. This interpretation is consistent with experimental evidence showing that sleep fragmentation can induce tissue-specific cytokine responses and that inflammatory and corticosterone responses may evolve differently over time (34, 35). Sleep disruption has also been shown to aggravate inflammation in experimental colitis (18).

Notably, corticosterone was increased in both DSS+LSF and DSS+HSF, whereas the Th2 increase was observed specifically in DSS+HSF. Therefore, the present data do not support a simple model in which greater fragmentation frequency produces proportionally greater stress-hormone and inflammatory responses. Instead, the MPO and Th2 findings further support domain-specific immune responses to different fragmentation frequencies. However, the underlying mechanisms remain uncertain and will require time-course analyses and functional assessment of individual immune-cell populations.

### 4.5 Microbiota and SCFA profiles differed across fragmentation conditions

Sleep disruption has been associated with alterations in gut microbial composition and fecal metabolite profiles (16, 17, 40). In the present study, alpha-diversity indices showed descriptive variation, with numerically higher Shannon diversity and species richness in DSS+HSF than in DSS+LSF, although corrected comparisons were not significant. In contrast, the weighted UniFrac distance metric was significantly higher in DSS+HSF than in DSS alone. Taxonomic analyses also identified distinct group-associated microbial signatures. These findings suggest that sleep-fragmentation conditions were associated with different microbial community profiles rather than a simple graded increase in dysbiosis.

Fecal SCFAs showed a partly different descriptive pattern. DSS+LSF tended to have lower acetate and propionate levels, whereas several SCFAs were relatively higher in DSS+HSF than in DSS+LSF. SCFAs are biologically relevant to epithelial metabolism, barrier maintenance, and immune regulation (9–11), and IBD has been associated with altered microbial functions and reduced abundance of selected butyrate-producing taxa (7, 8). Experimental studies have also shown that repeated sleep disruption can produce persistent changes in both the fecal microbiome and metabolome (41).

However, the SCFA patterns did not fully parallel the weighted UniFrac, immune, or barrier-associated findings. Because 16S rRNA sequencing does not directly establish microbial function, fecal SCFAs were measured only at the endpoint, and no microbiota-transfer or metabolite-intervention experiments were performed, these findings should be interpreted as associative and exploratory. Overall, the microbiota and SCFA results further support domain-specific responses to sleep-fragmentation frequency rather than a single linear microbiota–metabolite mechanism.

### 4.6 A potential mechanistic framework for the divergent endpoint patterns

One possible explanation for the non-parallel findings is that different biological domains respond to repeated sleep interruption with distinct temporal kinetics. Acute sleep fragmentation can activate hypothalamic–pituitary–adrenal signaling, increase corticosterone, and induce tissue- and time-dependent inflammatory responses (34, 35). In the present study, corticosterone was elevated in both DSS+LSF and DSS+HSF, but a single endpoint measurement cannot exclude earlier differences in the timing or magnitude of stress responses.

Within this framework, the higher MPO level and greater descriptive histological injury in DSS+LSF may reflect more prominent neutrophil-associated local inflammation, whereas the increased Th2 proportion and descriptive reductions in OCLN and TJP-1 in DSS+HSF may represent a different immune–epithelial response pattern. Experimental evidence suggests that glucocorticoid signaling during sleep fragmentation can modulate inflammation in a tissue- and context-dependent manner (42), while sympathetic signaling also contributes to sleep-fragmentation-associated inflammatory responses (43). Thus, different interruption frequencies may engage partially distinct neuroendocrine and inflammatory pathways rather than producing a uniform increase in disease severity.

Microbial and metabolic responses may constitute an additional component of this divergence. Repeated sleep disruption can induce persistent shifts in the fecal microbiome and metabolome (41), while SCFAs influence epithelial and immune homeostasis (9–11). However, because microbiota and SCFA patterns did not consistently parallel histological, immune, or barrier-associated findings, the present data do not establish a causal microbiota–SCFA–immune–barrier pathway. Overall, the observed dissociation among endpoints may reflect different response kinetics across stress, inflammatory, epithelial, and microbial–metabolic domains; this hypothesis requires longitudinal sampling and mechanistic validation.

### 4.7 Limitations and future directions

Several limitations should be acknowledged. First, sleep architecture was not directly measured by EEG/EMG; therefore, LSF and HSF represent apparatus-defined interruption frequencies rather than objectively quantified differences in sleep-stage disruption or arousal burden. This limitation is particularly relevant to the HSF protocol, in which frequent cage-floor sweeps may have introduced physical disturbance or stress-related effects in addition to sleep interruption. Although corticosterone was increased under both fragmentation protocols, locomotor activity, energy expenditure, and serial stress-hormone measurements were not assessed. Previous studies have shown that corticosterone and inflammatory responses to sleep fragmentation may follow distinct temporal trajectories (34, 35), underscoring the need for time-resolved physiological measurements.

Second, the endpoint design limits inference regarding the temporal sequence among histological injury, barrier-associated changes, immune responses, microbiota profiles, and SCFAs. In addition, 16S rRNA sequencing does not directly establish microbial function, and fecal SCFA concentrations were measured only at the endpoint. Thus, the observed microbiota and metabolite patterns should be considered associative. Longitudinal sampling, functional microbiome analyses, tissue metabolomics, SCFA supplementation, microbiota depletion, or fecal microbiota transfer will be required to test causal relationships.

Third, effective sample sizes varied across downstream assays because of sample availability and technical quality control, and several findings did not remain significant after multiple-comparison correction. These results should therefore be interpreted as descriptive or hypothesis-generating. The absence of an LSF-only group also limited assessment of the independent effects of low-frequency fragmentation in the absence of DSS.

Finally, only male mice were used, and potential sex-dependent responses were not evaluated. Future studies incorporating objective sleep measurements, longitudinal sampling, and targeted mechanistic interventions are needed to determine whether the observed domain-specific patterns reflect distinct biological adaptations to fragmentation frequency or different temporal stages of the response to sleep disruption.

## 5 Conclusion

In conclusion, low- and high-frequency sleep fragmentation were associated with distinct, endpoint-specific responses during DSS-induced colitis rather than a simple linear increase in disease severity. After multiple-comparison correction, both fragmentation protocols were associated with increased corticosterone, whereas MPO was more prominent in DSS+LSF and Th2 proportions and the weighted UniFrac distance metric were more evident in DSS+HSF. Histological injury, barrier-associated protein expression, inflammatory mediators, and fecal SCFA profiles showed additional descriptive differences across fragmentation conditions. Together, these findings suggest that sleep-fragmentation frequency may shape inflammatory, immune, microbial, metabolic, and barrier-related responses in a domain-specific manner. Further longitudinal and mechanistic studies incorporating objective sleep measurements are required to determine the causal relationships underlying these divergent response patterns.

## Data Availability

The 16S rRNA gene sequencing data presented in this study have been deposited in the NCBI Sequence Read Archive (SRA) under BioProject accession number PRJNA1516560: https://www.ncbi.nlm.nih.gov/bioproject/PRJNA1516560. The underlying numerical data supporting the figures and statistical analyses are available in the Zenodo repository: https://doi.org/10.5281/zenodo.21726586.
