# Supplementary material for: Sleep fragmentation frequency is associated with distinct immune, microbial, and barrier-related profiles in DSS-induced colitis in mice
Source: Front Immunol. 2026 Sep 15;17:1885856. doi: 10.3389/fimmu.2026.1885856 (PMC13619943; doi:10.3389/fimmu.2026.1885856)
Supplement: Supplementary file 1 [file Table1.docx]

**Supplementary Materials**

**Figure S1**: Daily food intake of mice. Daily food intake (g) was recorded across the experimental period.

**Figure S2**: Daily water intake of mice. Daily water intake (g) was recorded throughout the experimental period.
